# Transcriptome Analysis of the Larch Bud Gall Midge, *Dasineura rozhkovi* Mamaev et Nikolskij: De Novo Assembly, Phylogeny and Sexual Dimorphism

**DOI:** 10.3390/ijms27188089

**Published:** 2026-09-11

**Authors:** Maksim A. Deryuzhenko, Aleksandr V. Bobrovskikh, Olga D. Shishkina, Natalya V. Adonyeva, Olga V. Andreenkova, Natalja V. Shatskaya, Gennady V. Vasiliev, Nataly E. Gruntenko

**Affiliations:** Institute of Cytology and Genetics SB RAS, 630090 Novosibirsk, Russia; maksimd@bionet.nsc.ru (M.A.D.); avb@bionet.nsc.ru (A.V.B.);

**Keywords:** *Dasineura rozhkovi*, de novo transcriptome assembly, gall midges, phylogenetic analysis, sexual dimorphism

## Abstract

The larch bud gall midge (*Dasineura rozhkovi* Mamaev et Nikolskij) is one of the main pests infesting larches. Its activity causes developmental defects and a noticeable decrease in population growth in adult Siberian larches (*Larix sibirica* Ledeb.). Due to its complex life cycle and active presence in areas of human habitation, this insect is difficult to exterminate using common methods. *D. rozhkovi* has not been studied before with the use of sequencing technologies, which means there is no information on the mechanisms occurring in its body at the molecular level. Here, we present for the first time a de novo assembly and analysis of the *D. rozhkovi* transcriptome. We found a pronounced sexual dimorphism in differential gene expression in *D. rozhkovi* imagoes and performed a phylogenetic analysis using earlier-published genome assemblies of the insects belonging to several large clades. The resulting phylogenetic tree includes *D. rozhkovi* and eight other gall midge species and shows the closest relationship between *D. rozhkovi* and the Hessian fly (*Mayetiola destructor* Say). We believe that the obtained results can be further used for developing measures to control this pest.

## 1. Introduction

The larch bud gall midge *Dasineura rozhkovi* Mam. et Nik is a typical representative of the genus *Dasineura* of the family Cecidomyiidae, order Diptera [1]. So far, 6887 representatives of this family have been described; it is considered to be the largest among Diptera [2]. Cecidomyiidae includes six subfamilies: Catotrichinae, Lestremiinae, Micromyinae, Winnertziinae, Porricondylinae and Cecidomyiinae. *D. rozhkovi* is part of the largest subfamily, Cecidomyiinae; the majority of its representatives are phytophages, although there are also some mycophages and saprophages—and even zoophages [2]. Most phytophage larvae cause the formation of galls, inflations in plant tissues, inside which larval development takes place, hence the family name—the gall midges. These inflations occur when the original plant tissues are transformed under the action of gall midge larvae secretions. Most phytophagous gall midges only infest specific hosts, developing on one or two closely related plant species; the specialization of each gall midge species is so narrow that it pertains not only to the plant species but also to a specific organ of infestation [2,3]. Molecular phylogenetic studies of Cecidomyiidae conducted earlier [4,5] mostly support the commonly accepted morphological classification of this family [2]. However, it should be noted that in order to reconstruct the phylogenies, the authors used nine genetic markers, including both nuclear and mitochondrial genes in the first case and sequences of five mitochondrial and nuclear genes in the second case. Phylogenetic analysis of Cecidomyiidae at the full-genome or full-transcriptome level has not been performed previously. Here, we present for the first time the data on a de novo transcriptome assembly of *D. rozhkovi* imagoes and conduct a phylogenetic analysis using its proteome predicted based on the obtained transcriptome as well as the published genomes of eight other gall midge species, seven Diptera species of other families and seven insect species of other orders.

The larch bud gall midge is widespread in all of Russia and can often be found in populated areas where larches are planted [6,7]. The *D. rozhkovi* habitat spans Southern and Eastern Siberia up to Khabarovsk, as well as north-eastern Mongolia. The planted Siberian larches (*Larix sibirica* Ledeb.) suffer from this pest, in large part due to its narrow specialization and time of eclosion matching the period when the plants are most vulnerable to infestation.

**Figure 1:**
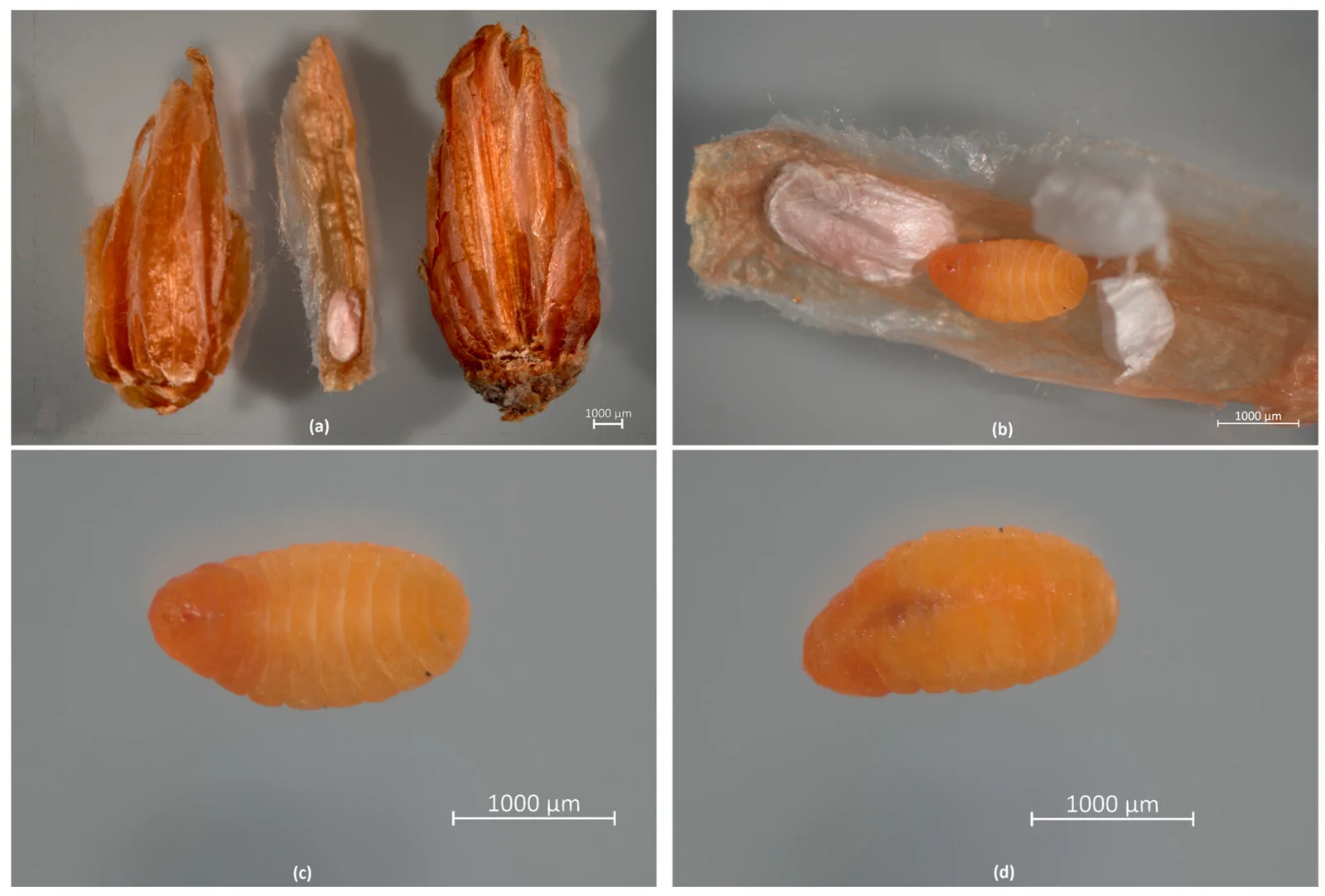
*Dasineura rozhkovi* larva: in a whitish pod located inside the bud gall removed from a *Larix sibirica* branch (**a**,**b**); ventral view (**c**); dorsal view (**d**).

*D. rozhkovi* spends its entire one-year life cycle on the branches of a single tree (not counting cases where an adult gall midge reaches the branches of a neighboring tree) [6,7]. They overwinter in galls on larch branches and emerge from them in early May. Female gall midges, using a long ovipositor, lay eggs in the bases of brachyblasts (shortened shoots) that begin to produce needles. Females lay 60–85 eggs during their lifetime [6]. Successful infestation depends directly on the brachyblast’s growth potential when the pest reaches the meristem. For each brachyblast, this period lasts no longer than three days (usually just one). Some buds are resistant to it, supposedly, due to a smaller growth potential and needle gain per day. Larch brachyblasts vary greatly in this parameter [8]. Developing larvae reach the growth apex where they begin to feed. Larval secretions modify bud morphology, leading to the creation of scales instead of needles, which results in the growth of a cone-shaped gall and the death of the brachyblast [9]. If a larva reaches the position too early or too late for the peak growth activation, an underdeveloped gall is formed. In the beginning of June, galls become noticeable [6]. A larva feeds in the gall and passes three stages during the summer [8]. Then, the larva forms a whitish pod under the outer scales of the gall, where it spends the winter (Figure 1a,b). Inside the pod the *D. rozhkovi* larva has a bright orange color (Figure 1c,d). In the spring, the larva pupates without leaving the pod, abandoning it a week later as an adult gall midge.

In a way, in an infested tree, galls take the place of brachyblasts. Gall midges fly out during the period of quick formation of bract scales. So, the bud formation of the tree and the life cycle of its parasite are perfectly synchronized. The gall midge causes changes leading to excess branching and the tree crown growing heavy. However, the most significant effect on the tree is undoubtedly caused by a decrease in its reproductive potential.

The gall size of *D. rozhkovi* can be used to distinguish it from another closely related species, *Dasineura kellneri* Henschel (Syn. *Dasineura laricis* F. Löw). The gall of *D. rozhkovi* is 0.9–1.4 cm in length, while that of *D. kellneri* is significantly smaller and round, rather than conical. The orange *D. kellneri* larva also overwinters in the gall and pupates there early in the spring of next year; this species is widespread in Central Europe and infests *Larix decidua* Mill [10].

**Figure 2:**
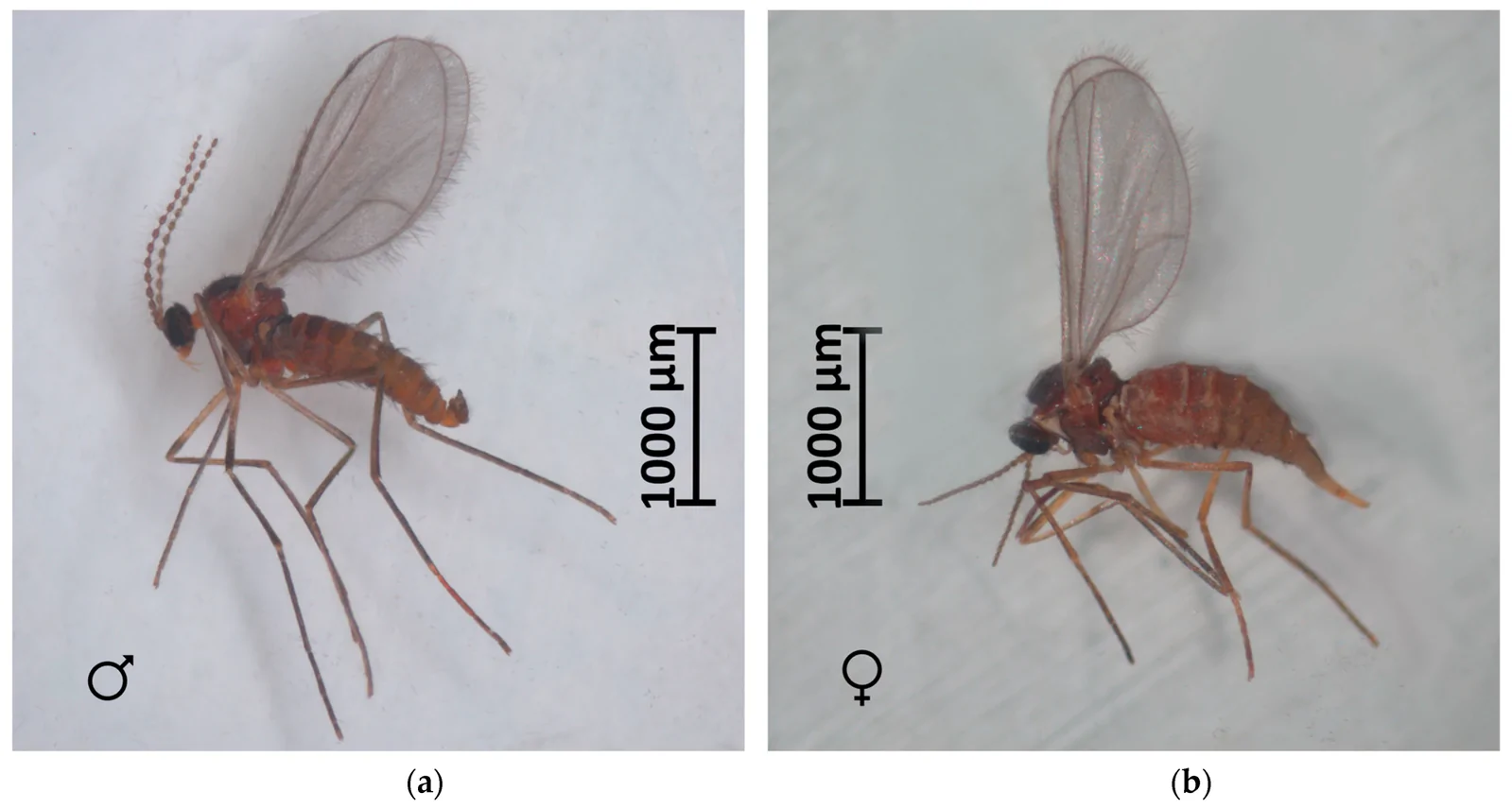
*Dasineura rozhkovi* imago: male (**a**) and female (**b**).

Imagoes of the Cecidomyiidae family representatives are usually very small (0.5–8 mm) and very fragile [10]. They have long thin legs and antennae, relatively large translucent wings with reduced venation and many hairs. *D. rozhkovi* are no exception (Figure 2). Males have longer antennae with larger segments and pronounced stems (Figure 2a). Females have a bigger abdomen, ending in a needle-like ovipositor (Figure 2b).

In a *D. rozhkovi* population, there are usually twice as many females as males [6]. Males live from 1 to 3 days; females live a little longer [6]. However, as of yet, the study of sexual dimorphism in gall midges has been mostly limited to morphological descriptions. In this work, we attempt for the first time to conduct a comparative analysis of males and females of one of the Cecidomyiidae species, *D. rozhkovi*, at the molecular level.

## 2. Results and Discussion

A de novo assembly of the gall midge *D. rozhkovi* transcriptome was obtained for the first time. In total, eight libraries were assembled: five for females and three for males.

According to the Trinity report, the primary de novo assembly comprised more than 298,000 transcripts and more than 170,000 genes (according to the Trinity terminology). We observed a significant redundancy, which could have resulted from allele variants, alternative splicing and/or transcript fragmentation. After reducing the redundancy via EvidentialGene (tr2aacds, release 2023.03.28), we obtained 46,268 transcripts and 36,552 proteins. Statistics for the resulting assembly are presented in Table S1.

The N50 and N90 values are sufficiently high (4135 bp and 1068 bp, respectively), indicating the quality of the resulting de novo assembly. The completeness of the assembly was assessed using BUSCO v5.7.1 in transcriptome and proteome mode against the insecta_odb10 lineage dataset (n = 1367). The analysis yielded the results displayed in Table 1.

The exceptionally high completeness score (98.1%) and minimal fragmentation (0.7%) confirm the high quality of our de novo reconstruction. The elevated proportion of duplicated complete BUSCOs (56.8%) is characteristic of transcriptome assemblies analyzed with the BUSCO v5.7.1 MetaEuk pipeline and primarily reflects retained allelic variants and alternative splicing isoforms rather than assembly redundancy, as further supported by the low duplication rate observed in proteome-level assessments.

We constructed gene coexpression networks reflecting the connections between genes differing in expression levels between females and males. A total of 10,619 genes were found to have sexual dimorphism at the expression level.

### 2.1. Phylogenetic Inference

**Figure 3:**
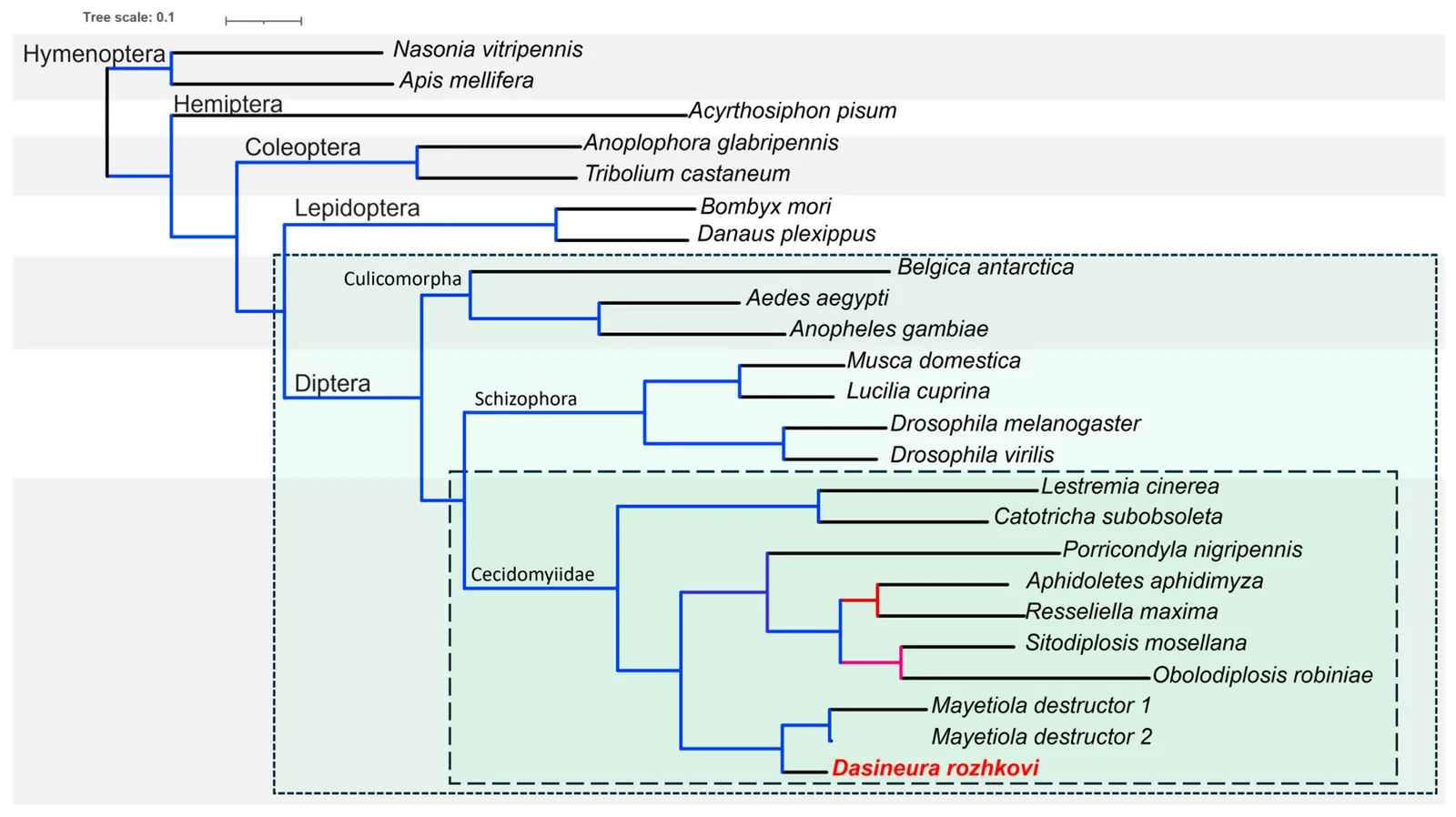
Insect phylogenetic tree reconstructed based on the orthogroups. Evolutionary divergence scale (upper left corner) corresponds to 0.1 substitutions per site. Branch colors reflect the level of bootstrap node support: blue—maximal support (BS = 1.0), pink/purple—moderate support (~0.7), red—low support (~0.4). The target species (*Dasineura rozhkovi*) is highlighted in red font. The dotted box included Diptera species, the dashed box included Cecidomyiidae species.

We constructed a phylogenetic tree based on protein-coding genes and including 16 Diptera species (Figure 3). To evaluate the speed of divergence for the representatives of the Cecidomyiidae family, we chose eight species from this family, for which genome data had been previously published, as well as whole-transcriptome data on *D. rozhkovi* obtained in the present study (Table 2). The tree also included seven other species belonging to the order Diptera: *Belgica antarctica* Jacobs (GCA_000775305.1); *Aedes aegypti* L. (GCA_002204515.1); *Anopheles gambiae* Giles (GCA_000005575.1); *Musca domestica* L. (GCA_030504385.2); *Lucilia cuprina* Wiedemann (GCA_022045245.1); *Drosophila melanogaster* Meigen (GCA_000001215.4 (Release 6)); and *Drosophila virilis* Sturtevant (GCA_030788295.1). Selected representatives of the order Hymenoptera (*Nasonia vitripennis* Walker (GCA_009193385.2) and *Apis mellifera* L. (GCA_003254395.2)) were used as an outgroup. For illustrative purposes, five representatives of the orders Hemiptera (*Acyrthosiphon pisum* Harris (GCA_005508785.2)), Coleoptera (*Anoplophora glabripennis* Motschulsky (GCA_000390285.2) and *Tribolium castaneum* Herbst (GCA_031307605.1)) and Lepidoptera (*Bombyx mori* L. (GCA_030269925.2) and *Danaus plexippus* L. (GCA_018135715.1)) were taken into analysis as well. The positions of these species on the phylogenetic tree determined in the course of analysis correspond to the current understanding of insect evolution.

### 2.2. Gene Regulatory Network of Highly Expressed Genes in Dasineura rozhkovi

**Figure 4:**
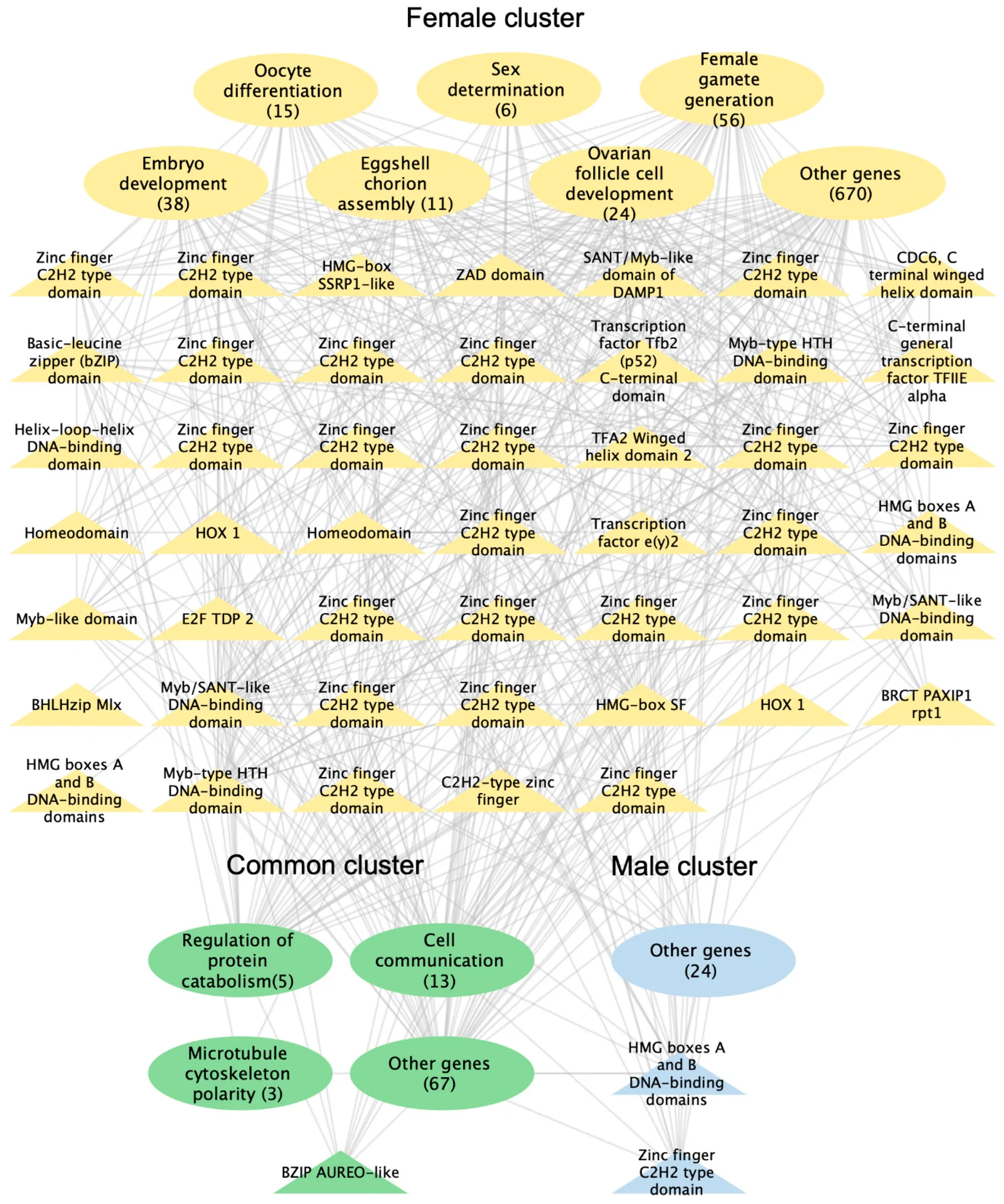
Network of processes and transcription factors associated with highly expressed genes in *Dasineura rozhkovi*. Female-related gene ontology processes for genes and transcription factors are marked in yellow, male-related gene group and transcription factors are marked in blue, and processes of genes highly expressed in both groups and the corresponding transcription factors are marked in green. Ellipses mark significantly enriched processes by gene clusters; triangles mark transcription factors. The number of genes in each category is shown in brackets.

In order to evaluate molecular genetic systems stably expressed in gall midge transcriptomes, we constructed a network of highly expressed genes. The image of this gene network, sorted by gene groups according to Gene Ontology terms, is presented in Figure 4. The entire gene network is provided in Supplementary Figure S1; its top 10 annotations can be found in Supplementary Figure S2; all annotations are located in Supplementary Tables S2 and S3. You can see which genes belong to which clusters by perusing Supplementary Table S4. The reconstructed gene network is based on gene coexpression and does not allow the direct establishment of cause-and-effect relations between regulatory interactions; however, it includes valuable biological insights described below.

In total, we found 47 transcription factors and 754 genes, mostly highly expressed in female transcriptomes. Almost half (21) of the transcription factors contain zinc finger C2H2-type domains. Myb-like/SANT-like (6), HMG box (4) and transcription factors with bZIP domains (2), homeodomains (2) and HOX 1 (2) are also present. This indicates the presence of complex machinery in transcription regulation of such specific female reproduction systems as oocyte differentiation, female gamete generation, embryo development, ovarian follicle cell development, eggshell chorion assembly and sex determination, significantly enriched in the group of target genes for these TFs.

Common genes highly expressed in both male and female transcriptomes (79 genes) are associated with such terms as cell communication, establishment or maintenance of microtubule cytoskeleton polarity and regulation of protein catabolic processes, which appear to be important for cellular and intercellular processes, as well as with the transcription factor BZIP AUREO-like.

Male transcriptomes seem to have no pronounced enrichment of functional terms in the gene subset (24); however, two specific TFs containing zinc finger C2H2-type and HMG box A/B domains have been found.

All in all, the network of highly expressed genes underlies the specificity of organization of female transcriptomes. The prevalence of C2H2 zinc fingers among female TFs agrees with the data on *Drosophila*, where the factors of this family potentially control oogenesis processes [11], and their functions are specific to Diptera [12]. High expression of two bZIP factors in the female transcriptome allows us to assume that they take part in chorion gene regulation, as has been shown for C/EBP in *D. melanogaster* [13], or that there is an association with ovary growth processes and vitellogenesis, as has been shown for the bZIP MafB factor in *Ae. aegypti* [14]. Myb/SANT-like domains (MADF) within the Mzfp1 protein in *D. melanogaster* are necessary for binding to housekeeping gene promoters and for recruiting the architectural protein CP190 [15]. In our study, we identified six transcription factors containing Myb/SANT- or Myb-like domains, which are significantly expressed in the female transcriptome of the gall midge. It can be assumed that these factors, like Mzfp1, take part in the regulation of genes involved in oogenesis, chorion formation and early embryo development. Earlier, it has been shown for *D. melanogaster* that the HMG-box proteins interact during spermatogenesis [16]; our work also shows their potential role in oogenesis processes.

It has been shown for the *Drosophila* genus that the expression of most genes is higher in males [17,18]; however, in the context of gene networks, we can see a strong female prevalence in gene expression in somatic tissues of the entire body in *D. rozhkovi*. This means that in insects, sexual specificity of expression may be organized in a more complex manner: males can have a larger total number of upregulated genes (5552 DEGs, 24 genes in the network), but strong coexpression networks are more characteristic of genes in females (5068 DEGs, 754 genes in the network).

Papa et al. [19] found that female-specific genes play a special role in four *Anopheles* species. Many of them are connected to reproductive tissues and are characterized by more rapid evolution and stricter selection compared to male-biased ones. The regulatory network we found in the gall midge reflects similar trends: it is female-biased, which underlies the consistency of transcription in oogenesis and female reproduction. Similar insights are described for *Ae. aegypti*: a faster evolution of the female reproductive system genes with a stronger selection pressure [20]. In addition, ovary development in *Ae. aegypti* is controlled by several endocrine axes, and oogenesis is described as a multi-step coordinated process demanding synchronous regulation of many genes [21]. In the context of the gene network we reconstructed, similar regulation patterns and a strict need for transcription consistency during oogenesis can also be seen.

So, *D. rozhkovi* was found to have a pronounced female-biased gene network organization, mostly connected to the regulation of oogenesis and other reproductive processes. The data obtained indicate that sexual dimorphism is defined not only by a set of differentially expressed genes but also by the particularities of their coordinated regulation, and the most complex transcription architecture is characteristic of the female transcriptome.

## 3. Materials and Methods

### 3.1. Insects

The branches of *L. sibirica* were collected and brought to the laboratory in March. The galls were cut from the branches with a scalpel and incubated in Petri dishes at 25 °C until the imagoes emerged. The hatched insects were separated by sex and frozen in liquid nitrogen.

### 3.2. RNA Isolation, cDNA Library Construction and RNA Sequencing

Three (for males) and five (for females) independent biological replicates were obtained by RNA extraction from whole insects (16–20 males or females per sample). The total RNA was extracted using TRI Reagent (T-9424, Sigma, St. Louis, MO, USA) according to the manufacturer’s instructions.

The quality of the RNA samples was evaluated using an Agilent Bioanalyzer 2100 (Agilent, Santa Clara, CA, USA). All the samples had RIN of 7.5 or higher. RNAseq library preparations were carried out with 1 μg of total RNA using a TruSeq Stranded mRNA Library Prep Kit and TruSeq RNA UD Indexes (Illumina, San Diego, CA, USA) according to the manufacturer’s instructions for barcoded libraries with minor modifications (6 min RNA fragmentation time and 10 PCR cycles were used). After normalization, the barcoded libraries were pooled and sequenced on a GenolabM sequencer (GeneMind, Shenzhen, China) 2 × 150 bp using GenoLab M Sequencing Set V4.0 (FCH 300 cycles).

### 3.3. De Novo Transcriptome Assembly

Quality assessment and read trimming were performed using Fastp v1.0 [22] with standard settings. Contig assembly was performed using the Trinity [23] program. The obtained assembly was analyzed using the built-in Trinity utilities.

Transcripts were also processed using the EvidentialGene (tr2aacds, release 2023.03.28) program in order to remove allele variants, combine isoforms and select high-quality transcripts. Redundancy was further reduced via clusterization at 95% identity using the CD-HIT (v4.8.1) program. Final transcripts, protein-coding and protein sequences were obtained. The completeness and quality of the transcriptome were assessed using BUSCO (v5.7.1) (the “insecta_odb10” set, n = 1367). The search for and the assignment of functional categories and orthologous groups, as well as the search for GO terms and KEGG pathways, were conducted using eggNOG-mapper (v2.1.12) on the entire database.

Domain and protein family annotation was conducted using InterProScan (v5.76-107.0).

### 3.4. Search for Differentially Expressed Genes

For differential expression analysis, trimmed Illumina reads were mapped to the final assembled transcriptome using RSEM v1.3.3 with Bowtie2 as the underlying aligner. The alignment was performed in paired-end mode with default parameters optimized for de novo assemblies. Differential expression analysis was performed at the gene level using the edgeR package (v3.40.0) in R. Raw expected counts generated by RSEM were imported directly into edgeR without prior normalization, as the software employs its own internal normalization methods robust to composition bias. A trimmed mean of M-values (TMM) normalization was applied to correct for library size differences and RNA composition effects. Dispersion estimation was conducted using the  estimateDisp  function, which fits a negative binomial generalized linear model (GLM) to account for biological variability between replicates. Statistical significance was assessed using the quasi-likelihood F-test ( glmQLFTest ), which provides more rigorous error rate control than the standard likelihood ratio test. Multiple testing correction was performed using the Benjamini–Hochberg procedure to control the false discovery rate (FDR). Genes were considered differentially expressed (DEGs) if they met the following thresholds: FDR-adjusted *p*-value < 0.05 and |log_2_ Fold Change| without restrictions.

This pipeline identified 10620 DEGs, comprising 5552 male-biased and 5068 female-biased genes.

### 3.5. Phylogenetic Tree Reconstruction

In order to reconstruct the phylogenetic tree, earlier-published genome assemblies of large-clade insects (including 8 gall midge species, Table 2) were used. For species with no published proteome, it was reconstructed based on the available genome assemblies. Proteome reconstruction was performed by the following method: genomes were translated into protein sequences via the ORF search without accounting for alternative splicing. To verify the results of this approach, the reconstructed *Mayetiola destructor* Say proteome was compared to the earlier-published one [24]. Using the OrthoFinder v2.5.5 program, based on protein sequences of 22 insect species belonging to large families (including 8 gall midge species), orthogroups were defined. Based on the obtained orthogroups, an evolutionary tree for 23 insect species (with representatives of the Hymenoptera order as an outgroup) was constructed.

### 3.6. Construction and Visualization of a Sex-Specific Gene Network

TPM-normalized expression values (transcripts per million) of 8 transcriptomic libraries (5 female, 3 male) were used as input data. In order to pick out genes with stable expression, the data were sorted in advance by the mean expression threshold ≥ 3 TPM and by the presence of such threshold expression in at least two samples. As a result, 13,301 genes were selected. The WGCNA algorithm (version 1.73) was used for constructing gene networks ([25]; https://cran.r-project.org/web/packages/WGCNA/index.html, accessed on 15 April 2026) in the R programming environment (version 4.5.3) (https://www.r-project.org, accessed on 15 April 2026). A standard coexpression analysis protocol with optimal parameters was used. In particular, expression matrices were separated into male and female subsamples for constructing sex-specific coexpression networks. Due to the small sample size, instead of the standard Pearson correlation, bicor correlation resistant to spikes was used. For each sex, degree values from 1 to 20 were tested using the pickSoftThreshold (corFnc = “bicor”, networkType = “signed”) function, and the degree closest to reaching scale-free network topology (R^2^ ≥ 0.8) was chosen. These were degree values 20 (for the female network) and 5 (for the male one). The details of the power selection procedure are provided in Supplementary Figure S3. For the male network (n = 3), we chose a more conservative power (5) to preserve biological signal, while for females (n = 5) a higher power (20) was selected to achieve a better scale-free fit (R^2^ = 0.722).

Next, adjacency matrices and topological overlap matrices (TOMs) were calculated. Complete TOMs contained about 88.5 million potential coexpression connections, making it impossible to draw a direct statistical inference regarding the significance of each connection. For this reason, a ranging approach was applied: 100,000 connections with greater TOM weights were taken from the entire matrix. They comprised approximately 0.06% of all possible gene pairs. This threshold allowed us to focus on more reliable coexpression interactions. To add potential regulatory interactions of these top connection subsets into the gene networks, connections comprising transcription factors (TF–gene or TF–TF connections) were chosen. A total of 9924 such connections were selected for the female subsample and 6033 for the male one.

We reconstructed a gene network corresponding to the following criteria:

1. Average log2 TMM gene expression in female and/or male groups ≥ 5;
2. TF–gene or TF–TF connections;
3. The connection being in the top 100,000 according to coexpression strength.

So, before the construction of the gene networks, a series of successive procedures was performed for filtering connections and genes in order to reveal the most relevant subnetworks associated with stable high expression or sexual dimorphism of the gall midge.

For further visualization of the gene networks, the Cytoscape program (version 3.10.3) was used ([26]; https://cytoscape.org, accessed on 15 April 2026). In particular, we used grid layout and node customization: transcription factors are shown as large triangles, and all other genes are shown as ellipses (the Shape, Height, and Width options). The node color demonstrates their sexual specificity (the Fill Color option). The Stroke Color option was used for rendering female and male network connections with different colors.

## 4. Conclusions

The data obtained demonstrate a pronounced sexual dimorphism in differential gene expression in *D. rozhkovi* imagoes. Phylogenetic analysis showed the closest relationship among the studied gall midges to be between the larch bud gall midge *D. rozhkovi* and the Hessian fly *M. destructor*.

## Figures and Tables

**Table 1:** BUSCO results.

Category	Transcriptomic Mode	Proteomic Mode
Complete (C)	1341 (98.1%)	1215 (88.8%)
Single-copy (S)	564 (41.3%)	1120 (81.9%)
Duplicated (D)	777 (56.8%)	95 (6.9%)
Fragmented (F)	10 (0.7%)	19 (1.4%)
Missing (M)	16 (1.2%)	133 (9.8%)
Total BUSCOs	1367	1367

**Table 2:** List of gall midge species used in phylogenetic analysis, with GenBank accession numbers.

Family	Subfamily	Species	Genome ID (NCBI Assembly)
Cecidomyiidae (gall midges)	Lestremiinae	*Lestremia cinerea*	GCA_027564135.1
	Catotrichinae	*Catotricha subobsoleta*	GCA_011634745.2
	Porricondylinae	*Porricondyla nigripennis*	GCA_026546635.1
	Cecidomyiinae	*Aphidoletes aphidimyza* *Resseliella maxima* *Sitodiplosis mosellana* *Obolodiplosis robiniae* *Mayetiola destructor* *Dasineura rozhkovi*	GCA_030463065.1 GCA_029041755.1 GCA_021018905.1 GCA_028476595.1 GCA_000149185.1

## Data Availability

The original data presented in the study are openly available in the NCBI database; the accession number is PRJNA1504075.

## Supplementary Materials

The following supporting information can be downloaded at https://www.mdpi.com/article/10.3390/ijms27188089/s1.
