# Supplementary material for: Transcriptome Analysis of the Larch Bud Gall Midge, Dasineura rozhkovi Mamaev et Nikolskij: De Novo Assembly, Phylogeny and Sexual Dimorphism
Source: Int J Mol Sci. 2026 Sep 11;27(18):8089. doi: 10.3390/ijms27188089 (PMC13606922; doi:10.3390/ijms27188089)
Supplement: Supplementary file 1 [file ijms-27-08089-s001.zip › Supplementary Table S1.pdf]

Table S1. Key metrics of the resulting build.

Indicator	Transcripts	Proteins
Number of sequences	46,268	36,552
Total length of the assembly	105,794,873 p.n. (~105.8 Mb)	9,969,520 aa (~9.97 Mb)
Minimum length	201 bp	30 aa
Average length	2,286.6 bp	272.7 aa
Maximum length	33,353 bp	10,640 aa
N50	4,135 bp	520 aa
N90	1,068 bp	107 aa
Median (Q2)	1,385 bp	125 aa
GC composition	35%	–
